# BUILDING SCIENTIFIC COLLABORATIONS IN ALZHEIMER’S DISEASE RESEARCH: THE ADRD SPECIAL INTEREST GROUP

**DOI:** 10.1093/geroni/igad104.1241

**Published:** 2023-12-21

**Authors:** Joseph Gaugler, Dana Urbanski, Hawking Yam, Zachary Baker, Emily Merkel, Stephen Waring, Kerry Sheets

**Affiliations:** University of Minnesota, Minneapolis, Minnesota, United States; University of Minnesota, Minneapolis, Minnesota, United States; University of Minnesota, Minneapolis, Minnesota, United States; Arizona State University, Tempe, Arizona, United States; University Of Minnesota, Minneapolis, Minnesota, United States; Essentia Institute of Rural Health, Duluth, Minnesota, United States; Hennepin Healthcare, Minneapolis, Minnesota, United States

## Abstract

Since the advent of the National Alzheimer’s Project Act, an unprecedented increase in federal funding to advance the science of dementia and dementia care has occurred. The Alzheimer’s Disease and Related Dementia (ADRD) Special Interest Group (SIG) of the University of Minnesota’s Center for Healthy Aging and Innovation aims to build and enhance scholarly collaborations in ADRD research between faculty, post-doctoral fellows, and graduate students across the University of Minnesota and beyond. Given the challenges of identifying mutual areas of scientific interest across disciplines, the ADRD has utilized several strategies to identify potential points of engagement, including a crosswalk procedure to create a matrix of members’ interests, methodological expertise, and available data resources; a member-led journal club; and a dashboard to track collaborative process. Although early in its development, the ADRD SIG aims to facilitate these initial partnerships to result in peer-reviewed publications and, eventually, team science-based project in ADRD.

